# DNA methylation analysis of aortic wall tissue in ascending aortic dilatation/aneurysm and dissection

**DOI:** 10.18699/vjgb-26-94

**Published:** 2026-10

**Authors:** I.A. Goncharova, A.A. Zarubin, A.V . Markov, S.A. Shipulina, N.P. Babushkina, Iu.A. Koroleva, A.A. Sleptcov, M.S. Nazarenko

**Affiliations:** Research Institute of Medical Genetics, Tomsk National Research Medical Center of the Russian Academy of Sciences, Tomsk, Russia; Research Institute of Medical Genetics, Tomsk National Research Medical Center of the Russian Academy of Sciences, Tomsk, Russia; Research Institute of Medical Genetics, Tomsk National Research Medical Center of the Russian Academy of Sciences, Tomsk, Russia; Research Institute of Medical Genetics, Tomsk National Research Medical Center of the Russian Academy of Sciences, Tomsk, Russia; Research Institute of Medical Genetics, Tomsk National Research Medical Center of the Russian Academy of Sciences, Tomsk, Russia; Research Institute of Medical Genetics, Tomsk National Research Medical Center of the Russian Academy of Sciences, Tomsk, Russia; Research Institute of Medical Genetics, Tomsk National Research Medical Center of the Russian Academy of Sciences, Tomsk, Russia; Research Institute of Medical Genetics, Tomsk National Research Medical Center of the Russian Academy of Sciences, Tomsk, Russia

**Keywords:** ascending aortic dilatation/aneurysm, ascending aortic dissection, DNA methylation, HEpiDISH, cell-type adjustment, дилатация/аневризма восходящей аорты, расслоение восходящей аорты, метилирование ДНК, HEpiDISH, поправка на клеточный состав

## Abstract

Ascending aortic dilatation/aneurysm and dissection are life-threatening cardiovascular conditions. However, the epigenetic regulatory mechanisms within aortic cells underlying these pathologies remain poorly understood. This study aimed to identify differentially methylated genes (DMGs) in aortic tissues from patients with ascending aortic dilatation/aneurysm and dissection by performing a re-analysis of publicly available DNA methylation datasets. A re-analysis was conducted on three DNA methylation datasets (GSE50192, GSE202047, GSE84274) from the Illumina Infinium HumanMethylation450 BeadChip. To account for tissue heterogeneity, cellular deconvolution was performed using the HEpiDISH algorithm and a custom reference panel of cell-type-specific CpG sites for key vascular cell types (endothelial cells, fibroblasts, smooth muscle cells, immune cells). Our re-analysis revealed that aortic dissection and bicuspid aortic valve patients with aortic dilatation/aneurysm exhibit distinct DNA methylation profiles compared to intact aorta. These pathologies differ from each other in the number and the spectrum of DMGs. The adjustment for cellular heterogeneity eliminated the majority of the initially identified DMGs. However, this correction helped to make an assessment of vascular inflammation, characterize its impact on aortic wall stability, and identify genes with differential DNA methylation associated with the disease phenotypes, rather than with inter-group heterogeneity. The genes NFIX, PCDH7, MED13L, LMO4, DIP2C, PLEKHF1, NKD1, C10orf41 (ZNF503-AS2), ARAP2, TBX3, and TBX5, which remained in the DMG status after adjustment for cellular composition and are expressed in individual cells/whole aortic tissue, can be considered as new candidate genes for the development of aortic dilatation/aneurysm in patients with bicuspid aortic valve. NFIX, LMO4, DIP2C, C10orf41 (ZNF503- AS2), MED13L, TBX3, TBX5 are transcriptional regulators or modulators; NKD1 is a recognized inhibitor of the Wnt pathway. Our findings highlight the necessity of a rigorous approach to cohort design, which includes comprehensive phenotyping (encompassing aortic valve morphology) and underscore the importance of future single-cell investigations.

## Abstract (ru)

Дилатация/аневризма и расслоение восходящей аорты относятся к жизнеугрожающим заболеваниям, и выявление эпигенетического ландшафта, регулирующего функционирование генома в клетках аорты при данной патологии, является актуальной задачей. В связи с этим в настоящем исследовании выполнена идентификация дифференциально метилированных генов (ДМГ) в тканях аорты при дилатации/аневризме и расслоении восходящего отдела с помощью реанализа данных по метилированию ДНК, представленных в открытом доступе в репозитории NCBI GEO (Gene Expression Omnibus). Проведен реанализ результатов исследований (GSE50192, GSE202047, GSE84274), реализованных на платформе Illumina Infinium HumanMethylation450 BeadChip. Биоинформатическая деконволюция клеточного состава тканей сосудов осуществлена с помощью алгоритма HEpiDISH, а также разработанного референса, включавшего набор CpG-сайтов, индекс метилирования которых специфичен для эндотелиальных клеток, фибробластов, гладкомышечных и иммунных клеток. Реанализ данных показал, что расслоение аорты и ее дилатация/ аневризма с двухстворчатым аортальным клапаном характеризуются специфическим профилем метилирования ДНК относительно интактной ткани аорты, поскольку различаются как по числу, так и по спектру ДМГ. Поправка на клеточный состав аннулирует основное количество ДМГ, но позволяет составить представление о наличии воспаления в ткани аорты, оцениваемого по степени инфильтрации ткани иммунными клетками, охарактеризовать роль воспалительного инфильтрата в нарушении целостности стенки аорты и выявить гены, отражающие различия, связанные с изучаемыми фенотипами, а не с межгрупповой гетерогенностью клеточных популяций. Гены NFIX, PCDH7, MED13L, LMO4, DIP2C, PLEKHF1, NKD1, C10orf41 (ZNF503-AS2), ARAP2, TBX3, TBX5, оставшиеся в статусе ДМГ после поправки на клеточный состав и экспрессируемые в отдельных клетках/цельной ткани аорты, могут рассматриваться как новые гены-кандидаты развития дилатации/аневризмы аорты у пациентов с двухстворчатым аортальным клапаном. Белковые продукты генов NFIX, LMO4, DIP2C, C10orf41 (ZNF503-AS2), MED13L, TBX3 и TBX5 являются транскрипционными факторами или регуляторами транскрипционной активности ДНК, а гена NKD1 – ингибитором сигнального пути Wnt. Полученные данные подчеркивают необходимость строгого подхода к формированию групп сравнения с полным описанием исследуемого фенотипа, включающим морфологию аортального клапана, и проведения исследований на уровне отдельных клеток.

## Introduction

DNA methylation is one of the main mechanisms of epigenetic modifications of the genome, both under normal conditions and in pathology. Nevertheless, studies investigating DNA methylation in pathologically altered tissues from ascending aortic dilatation/aneurysm and dissection are scarce (Shah et al., 2015; Pan et al., 2017; Björck et al., 2018; Li N. et al., 2020; Liu et al., 2021; Chen Y. et al., 2022; Goncharova et al., 2024; Koroleva et al., 2024). The data obtained from these studies show little overlap, which is due to phenotypically heterogeneous patient groups, different tissues (fragments of various aortic segments, peripheral blood) used in the studies, and different methods for DNA methylation assessment. Nonetheless, a comprehensive analysis of the results permits several conclusions, including the following: differentially methylated regions between diseased and intact aortic tissue are enriched in genes implicated in cardiovascular system development; the MAPK, WNT, TGFβ, and HOX signaling pathways participate in the pathogenesis of aortic aneurysm; and aortic valve morphology constitutes a critical determinant of the pathogenetic mechanisms underlying ascending aortic dilatation/aneurysm progression.

It is well established that cellular heterogeneity constitutes one of the most significant factors influencing the outcomes of DNA methylation studies. The walls of blood vessels contain varying proportions of several cell types that comprise their layered structure, as well as immune cells. All of these cells exhibit distinct DNA methylation patterns. Hence, alterations in DNA methylation patterns may reflect changes in cellular composition rather than provide reliable information regarding aberrant DNA methylation. In the present study, we consider arterial cell composition as a variable that affects DNA methylation levels

Therefore, the aim of the present study was to identify differentially methylated genes in diseased aortic tissues in ascending aortic dilatation/aneurysm and dissection, while adjusting for cell composition, by integrating and re-analyzing publicly available DNA methylation data from pathologically altered and control tissues of the ascending aorta deposited in the NCBI Gene Expression Omnibus (GEO) repository.

## Materials and methods

We re-analyzed publicly available results of studies GSE50192, GSE202047, and GSE84274, which were performed using the Illumina Infinium HumanMethylation450 BeadChip platform.

A description of the studies GSE50192, GSE202047, and GSE84274, that were used for the re-analysis, is presented in Table 1. The samples analyzed in GSE50192 comprised autopsy material from seventeen somatic tissues obtained from four individuals: three males, two of whom died from myocardial infarction (aged 40 and 53 years) and one who died from hemorrhagic stroke at 54 years of age; and one female, who died from hemorrhagic stroke at 60 years of age. For subsequent analyses, we selected DNA methylation data from samples of coronary arteries, as well as from the ascending and abdominal aorta.

**Table 1.**
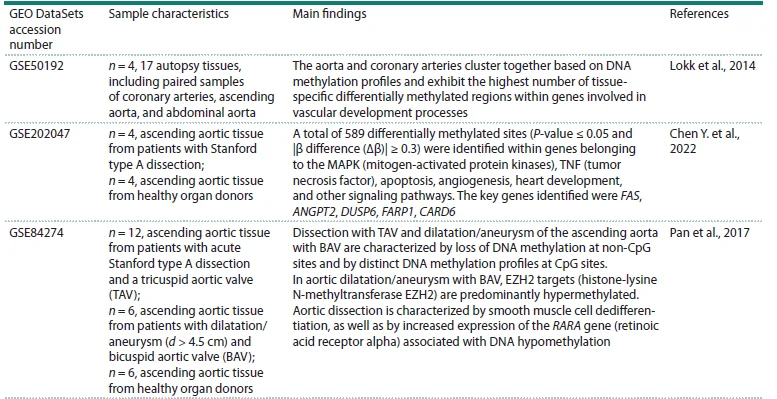
Characteristics of the studies included in the re-analysis of DNA methylation data from tissues of the ascending and abdominal aorta, coronary arteries, and pathologically altered tissues of the ascending aorta

The samples included in GSE202047 are ascending aortic tissues from patients with Stanford type A dissection (TAAD) (n = 4) and from healthy organ donors (n = 4), hereinafter referred to as intact ascending aorta (IAA). Both patients and healthy donors were male, with mean ages of 49.10 ± 4.91 years and 47.92 ± 6.73 years, respectively.

In GSE84274, we analyzed ascending aortic tissue samples from patients with acute Stanford type A dissection and a tricuspid aortic valve (n = 12; 10 males (52.4 years), 2 females); from patients with aortic dilatation/aneurysm (d > 4.5 cm) and bicuspid aortic valve (TAA with BAV) (n = 6; 5 males (53.8 years), 1 female); and from healthy organ donors (IAA) (n = 6; all males, 36.7 years).

Statistical analysis of the data was performed using the lumi and limma packages within the R software environment (Bioconductor). Principal component analysis (PCA) was employed for dimensionality reduction. Correction for false discovery rate (FDR) in multiple comparisons was performed using the Benjamini–Hochberg method. β-values were used to assess DNA methylation levels.

CpG sites (DMS) were considered differentially methylated if they exhibited a difference in mean methylation levels between sample groups with FDR <0.05 and |Δβ| ≥ 0.2. Genes containing DMS were defined as differentially methylated genes (DMGs). The first twenty sites with the greatest differences in DNA methylation level between the compared tissues, as determined by the |Δβ| value, were characterized in detail.

Identification of DMS in ascending aortic pathology with adjustment for cell composition was performed using the HEpiDISH algorithm for deconvolution of DNA methylation profiles (Hierarchical Epigenetic Dissection of Intra-Sample- Heterogeneity; Zheng et al., 2019), as well as a reference panel previously developed by our group and adapted for blood vessel tissues. This reference panel includes a set of CpG sites with methylation specific to endothelial cells (datasets GSE84395, GSE122126, GSE82234, GSE40699 from the GEO repository), fibroblasts (GSE40699), smooth muscle cells (SMCs) (GSE40699, GSE113061), immune cells (GSE35069), and the data ENCSR420WUN from the study ENCODE (Markov et al., 2020).

Functional annotation of the identified DMGs was performed using the gene expression database for aortic arch tissues (GTEx Portal; https://gtexportal.org), single-cell transcriptomic data from studies on ascending aortic aneurysm (PlaqView; https://plaqviewv2.pods.uvarc.io), gene expression data for ascending aortic aneurysm publicly available in the Gene Expression Omnibus database (GSE83675, GSE61128, GSE5180), as well as data from the scientific literature (Kim et al., 2010; Doppler et al., 2023; Huang M. et al., 2023).

We performed a comparative analysis between the set of DMGs and genes associated with coronary artery disease (CAD), myocardial infarction (MI), and bicuspid aortic valve (BAV) based on genome-wide association studies (GWAS) available in the GWAS Catalog database (https://www.ebi. ac.uk/gwas), as well as genes implicated in hereditary and syndromic forms of thoracic aortic aneurysm (Elbitar et al., 2021; Li Y. et al., 2021).

## Results

Analysis of CpG site methylation profiles in aortic samples within the integrated dataset revealed that autopsy arterial samples (GSE50192) distinctly separate from arterial tissues collected during surgical treatment (GSE202047 and GSE84274) (Fig. 1).

**Fig. 1.**
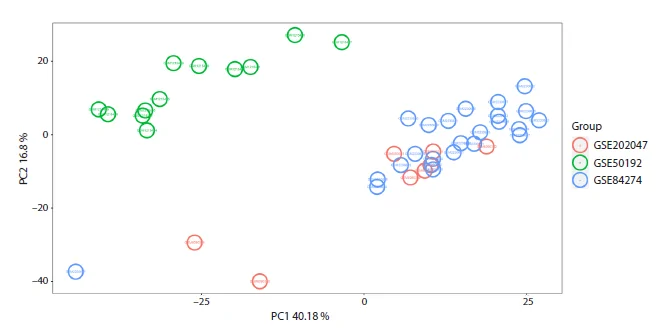
Distribution of the analyzed samples in the space of the first two principal components according to the methylation level of CpG sites presented in GSE50192, GSE202047, and GSE84274.

Therefore, only the data from GSE202047 and GSE84274 were combined, including all samples. The data obtained from study GSE50192 were not analyzed further.

**Identification of differentially methylated DNA sites in ascending aortic tissues: dilatation/aneurysm with valve anomaly and thoracic aortic dissection compared to intact aortic tissue**

Comparative analysis of DNA methylation levels between tissues of TAA with BAV and IAA revealed 57 DMS (14 DMGs). Among all DMS, 22 were localized in CpG shores and 14 in CpG islands (Table 2; sheet “TAA with BAV_IAA” in the Supplementary Materials)1. The majority of DMS (84.2 %) were hypermethylated in TAA with BAV tissues relative to IAA.

**Table 2.**
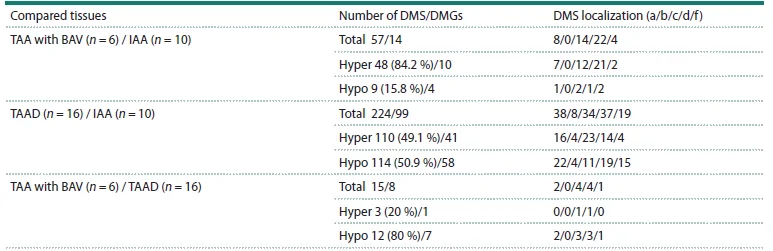
Characteristics of differentially methylated CpG sites/genes in the compared groups Note. BAV – bicuspid aortic valve, IAA – intact ascending aorta, TAA – thoracic aortic dilatation/aneurysm, TAAD – thoracic aortic aneurysm and dissection; DMS localization: a – promoter (TSS, 5’-UTR, 1st exon); b – 3’-UTR; c – CpG island; d – CpG shore (less than 2 kb from a CpG island); f – CpG shelf (2–4 kb from a CpG island).

Supplementary Materials are available in the online version of the paper: https://vavilov.elpub.ru/jour/manager/files/Suppl_Gonchar_Engl_30_6.xlsx


Within the nucleotide sequences of annotated genes, 23 CpG sites were identified, eight of which were localized in promoters. Among the 23 CpG sites located within the sequences of known genes, the hypomethylated sites included those in the DIP2C (Δβ = –0.29), TMEM132C (Δβ = –0.29; Δβ = –0.25), CD82 (Δβ = –0.23), and ZFPM1 (Δβ = –0.22) genes, with the CD82 CpG site (cg01889574) being located in the promoter. All of these hypomethylated DMS/DMGs, with the exception of ZFPM1 (cg06099697), were among the top 10 sites with the greatest differences in DNA methylation levels between the compared tissues (sheet “TAA with BAV_IAA” in the Supplementary Materials).

The remaining DMGs are characterized by the presence of CpG sites hypermethylated in TAA with BAV, with two DMS identified in each of the ISL1 and KCNN2 genes, and six DMS in the TBX5 gene (sheet “TAA with BAV_IAA” in the Supplementary Materials). Among the top CpG sites with increased methylation levels in diseased aortic tissue were ISL1 (Δβ = 0.32; Δβ = 0.25), SNX25 (Δβ = 0.31), and TBX5 (Δβ = 0.26; Δβ = 0.26; Δβ = 0.25). Site cg12836958 and two sites, cg23820885 and cg09042277, are located in the promoters of the SNX25 and TBX5 genes, respectively (sheet “TAA with BAV_IAA” in the Supplementary Materials).

Comparison of DNA methylation levels between ascending aortic tissues with dissection (TAAD) and IAA revealed 224 DMS (99 DMGs). Among all DMS, 37 were localized in CpG shores and 34 in CpG islands (Table 2; sheet “TAAD_ IAA” in the Supplementary Materials). 130 CpG sites were identified within the nucleotide sequences of 99 genes, among them 38 CpG sites were localized in promoters. Hypermethylated DMS in TAAD samples accounted for 49.1 % of all DMS. Among the top 10 CpG sites, only those in the ADARB2 (Δβ = 0.37; Δβ = 0.35) and TTLL8 (Δβ = 0.28) genes were hypermethylated. Two hypomethylated sites, cg07162000 (Δβ = –0.24) and cg17861863 (Δβ = –0.23), were also identified in the ADARB2 gene.

Among the top 10 CpG sites, four sites of the TBX1 gene (Δβ = –0.34 to –0.29), located within a CpG island, as well as sites of the FLJ42709 (NR2F1-AS1) (Δβ = –0.32), COL21A1 (Δβ = –0.29), and MAP3K5 (Δβ = –0.29) genes, were hypomethylated in TAAD samples (Table 2; sheet “TAAD_IAA” in the Supplementary Materials).

Comparison of DNA methylation levels between TAA with BAV and TAAD tissues revealed 15 DMS (8 DMGs). Among all DMS, four were localized in CpG shores and five in CpG islands. In the CYP2W1 and PLEKHF1 genes, two CpG sites (cg09414673 and cg15561501, respectively) are located in the promoter (Table 2; sheet “TAA with BAV and TAAD” in the Supplementary Materials). Fifteen (80 %) of all DMS were hypomethylated in diseased aortic tissues from patients with TAA and BAV relative to aortic dissection (Table 2; sheet “TAA with BAV and TAAD” in the Supplementary Materials).

Among the CpG sites located within known genes, the greatest differences in methylation levels between the compared tissues were observed for sites hypomethylated in TAA with BAV tissues, specifically those in the DIP2C (Δβ = –0.27) and NFIX (Δβ = –0.27; Δβ = –0.24) genes. Two sites, cg15561501 (PLEKHF1) and cg09414673 (CYP2W1), located in gene promoters, were also hypomethylated in TAA with BAV tissues (Δβ = –0.21 each; sheet “TAA with BAV and TAAD” in the Supplementary Materials). A DMS in the SLC1A3 gene was hypermethylated in diseased aortic tissues from patients with TAA and BAV (Δβ = 0.21).

**Reconstruction of the cellular composition of ascending aortic tissues in dilatation/aneurysm with valve anomaly, aortic dissection, and intact aortic tissue**

Since tissue cellular composition is a significant factor for determining DMS (Houseman et al., 2015, 2016), bioinformatic deconvolution of the cellular composition of aortic tissues was also performed during data analysis (Fig. 2).

**Fig. 2.**
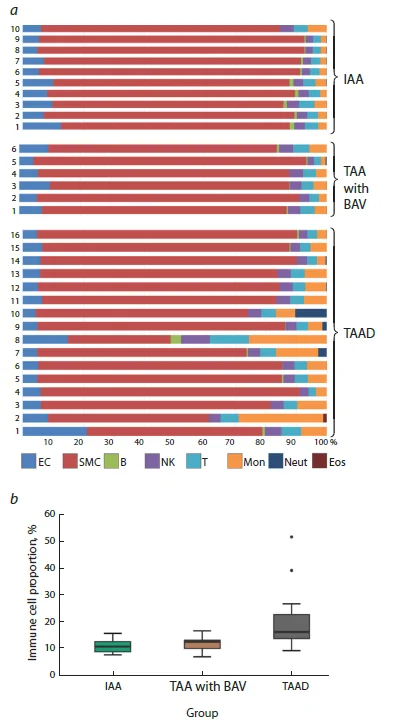
Reconstruction of the cellular composition of the ascending aorta under normal and pathological conditions using the HEpiDISH algorithm and the developed methylome reference panel for vascular cells. a – distribution of cell types in ascending aortic tissues in the studied groups (the vertical axis indicates the sample number within each group). EC – endothelial cells, SMC – smooth muscle cells, B – B lymphocytes, NK – natural killer cells, T – T lymphocytes, Mon – monocytes, Neut – neutrophils, Eos – eosinophils; b – proportion of immune cells in the studied sample groups according to the results of cellular composition assessment.

Application of the DNA methylation prof﻿ile deconvolution algorithm to predict the proportions of expected cell types in ascending aortic tissues revealed that smooth muscle cells (SMCs) (33.7–88.7 %) and the leukocyte component (6.7–51.3 %) constitute a substantial proportion of cells, while vascular endothelial cells (ECs) are present to a lesser extent (4.2–21.2 %), and fibroblasts were not detected by the algorithm. Among leukocytes, monocytes account for a significant fraction (1.4–27.8 %).

The proportion of immune cells in IAA does not exceed 15 % (10.7 % on average). In TAA with BAV, this value ranges from 6 to 16 % (11.6 % on average). In TAAD, immune cell infiltration into the vascular wall ranges from 9 to 51 %, with a mean of 19.6 %, which is significantly higher than that in the intact aorta (p = 0.003) (Fig. 2).

**Identification of DMS and their host genes in ascending aortic pathology with adjustment for cell composition**

After adjusting for cell composition, the list of CpG sites hypomethylated in TAA with BAV samples compared to IAA retained only site cg26561082 of the DIP2C gene (Δβ = –0.29), located in a CpG shore region (Table 3).

**Table 3.**
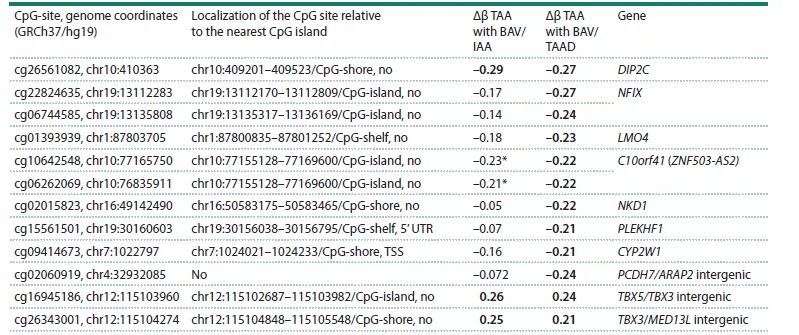
Differentially methylated CpG sites between the studied groups after adjustment for cell composition Note. Values of Δβ for CpG sites meeting the DMS criteria (FDR < 0.05 and |Δβ| ≥ 0.2) are shown in bold; * Δβ for CpG sites with FDR > 0.05; TSS, 5’ -UTR – promoter.

Comparison of DNA methylation levels with adjustment for cell composition between the TAAD and IAA groups revealed no significant differences.

Comparison of DNA methylation levels between TAA with BAV and TAAD after adjustment for cell composition revealed 12 DMS, of which two were hypermethylated and ten were hypomethylated in the aorta with dilatation/aneurysm (Table 3). Seven differentially methylated genes (LMO4, NFIX, CYP2W1, C10orf41 (ZNF503-AS2), PLEKHF1, DIP2C, NKD1) contained hypomethylated CpG sites in TAA with BAV relative to aortic dissection (Table 3).

Furthermore, three CpG sites were located in intergenic regions: cg02060919 (PCDH7/ARAP2) was hypomethylated in the TAA with BAV group compared to TAAD, while sites cg16945186 (TBX5/TBX3) and cg26343001 (TBX3/MED13L) were hypermethylated in ascending aortic dilatation/aneurysm relative to both aortic dissection and intact aortic tissue.

Thus, after adjustment for cell composition, TAA with BAV compared to both IAA and TAAD shows decreased methylation levels of the majority of identified DMGs. These include genes encoding functionally active molecules (transcription factors DIP2C, LMO4, NFIX; lncRNA C10orf41 (ZNF503- AS2)), an inhibitor of the Wnt signaling pathway (NKD1), as well as genes whose protein products mediate apoptosis and autophagy (PLEKHF1), focal adhesion and intercellular interactions (PCDH7, ARAP2), or belong to the cytochrome P450 family (CYP2W1).

Elevated methylation levels were observed for intergenic CpG sites, which likely influence the functional activity of the transcription factor genes TBX3 and TBX5, as well as the MED13L gene, whose protein product belongs to the mediator complex family (MED), a complex that plays a key role in the transcription of numerous eukaryotic genes.

**Functional annotation of DMGs using databases and published studies**

Analysis of expression of the DMGs identified after cell composition adjustment in aortic arch tissues according to the GTEx Portal data showed that, among the aforementioned genes, NFIX (131.7 TPM – transcripts per million) and PCDH7 (65.4 TPM) exhibited the highest expression levels. The genes MED13L, LMO4, DIP2C, PLEKHF1, and NKD1 were expressed at moderate levels (32.3; 29.6; 28.9; 27.1; and 16.5, respectively), whereas C10orf41 (ZNF503-AS2), TBX3, ARAP2, TBX5, and CYP2W1 showed almost no expression in the aortic arch (0.06–3.9 TPM).

Analysis of gene expression data for ascending aortic aneurysm in patients with BAV and TAV, available from public repositories (GSE5180, GSE83675, GSE61128 from the GEO repository) and scientific publications (Kim et al., 2010; Doppler et al., 2023), led to the conclusion that the DMGs identified in the present study do not significantly alter their functional activity either in the diseased aorta relative to intact tissues, or in dilated aortic tissues from patients with different aortic valve anatomy.

According to data from the PlaqView resource, in aortic tissue from 11 individuals, including eight patients with ascending aortic aneurysm and three healthy individuals (Li Y. et al., 2020), the NFIX and LMO4 genes are expressed in a substantial number of SMCs and fibroblasts, with high expression levels for NFIX and approximately half that for LMO4 (Fig. 3). Functional activity of the PCDH7 gene was detected at high levels in a large percentage of SMCs and, to a lesser extent, in fibroblasts. The DIP2C gene is expressed at moderate levels, but in a small number of SMCs, fibroblasts, and mast cells. ARAP2 activity was detected in T, B, and NK cells. The MED13L gene is expressed in a wide range of cells at moderate levels. The remaining DMGs are expressed in no more than 20 % of cells in ascending aortic tissues (Fig. 3).

**Fig. 3.**
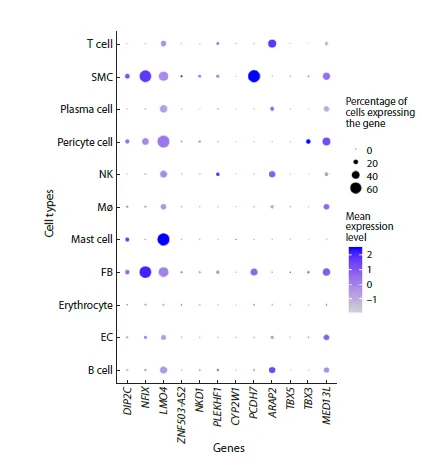
Cell types and mean relative expression levels of the DMGs identified in the present study according to the PlaqView resource, in a single-cell dataset of the ascending aorta from 11 individuals, including eight patients with ascending aortic aneurysm and three healthy individuals (Li Y. et al., 2020). DMG – differentially methylated genes. SMC – smooth muscle cells; NK – natural killer cells; Mø – macrophages; FB – fibroblasts; EC – endothelial cells.

Comparative analysis of the DMGs with lists of genes associated with coronary artery disease (CAD), thoracic aortic aneurysm based on GWAS results, bicuspid aortic valve, or genes implicated in hereditary aortic aneurysms revealed that rs543659530 in the PCDH7 gene, encoding protocadherin 7, is associated with CAD in patients with type 2 diabetes mellitus (T2DM) (Song et al., 2021), and rs61937394 in the MED13L gene, whose protein product belongs to the mediator complex involved in transcription regulation, is associated with the development of ascending aortic aneurysm (Pirruccello et al., 2022). The remaining DMGs were not present in any of these lists.

## Discussion

The use of a DNA methylation profile deconvolution algorithm and cellular composition assessment with a customized reference panel for vascular tissues allowed us to conclude that smooth muscle cells and immune cells constitute a substantial proportion of cells in the ascending aorta. The degree of immune cell infiltration into the vascular wall in ascending aortic dissection significantly exceeds that observed in intact vascular tissues.

Sun Pan et al. (2017) also performed adjustment for cell composition, albeit using a different algorithm (Houseman CP). The authors observed a decrease in the mean number of SMCs and an increase in the number of ECs in aortic dissection compared to normal aorta and dilated aorta/aneurysm with BAV (Pan et al., 2017). In the present study, the mean numbers of SMCs and ECs did not differ between groups, whereas immune cell infiltration was higher in aortic dissection. This finding may indicate a role of the inflammatory infiltrate in disrupting the integrity of the aortic wall.

It should also be noted that, according to the deconvolution algorithm results, fibroblasts were not detected in the studied samples, despite their expected abundance based on generally accepted knowledge of aortic wall structure.

Numerous studies employing transcriptome and epigenome analysis technologies in single cells from human artery samples and animal models have demonstrated that SMCs possess extensive phenotypic plasticity and, together with fibroblasts and pericytes, typically constitute a single supercluster of cells originating from mesenchymal stem cells (Mosquera et al., 2023; Zhao et al., 2025).

Thus, we believe that the “SMC component” in the deconvolution results may represent a mixture of different cell types (with the exception of pericytes) that are similar in their gene expression profiles and, apparently, DNA methylation patterns, including a spectrum of differentiated and modulated SMCs, as well as fibroblasts

The re-analysis of DNA methylation data in aortic tissues revealed that dilatation/aneurysm with BAV and ascending aortic dissection exhibit distinct DNA methylation statuses relative to intact aortic tissue, as these phenotypes differ from each other both in the number and in the spectrum of DMGs. However, adjustment for cell composition eliminates the majority of DMS, leaving only one CpG site in the DIP2C gene and two sites in the intergenic regions TBX5/TBX3 and TBX3/MED13L when comparing aortic dilatation/aneurysm with BAV and intact tissue. Comparison between dilatation/aneurysm and aortic dissection revealed ten differentially methylated CpG sites in the genes DIP2C, LMO4, NFIX, C10orf41 (ZNF503- AS2), PLEKHF1, NKD1, CYP2W1, PCDH7/ARAP2, TBX5/ TBX3, and TBX3/MED13L.

The obtained results likely reflect differences in DNA methylation patterns associated with sample cellular composition variability to a greater extent than significant epigenetic differences between diseased and intact aortic tissues. This is supported by earlier studies of gene expression conducted on different tissue types (intima-media and adventitia of the aorta, as well as intima-media of the internal thoracic artery), which demonstrated that between 22.5 and 52 % of all genes are differentially expressed in pairwise tissue comparisons (Folkersen et al., 2011).

Furthermore, the Gene Expression Omnibus data used for the re-analysis did not specify the aortic valve morphology in patients with dissection (GSE202047), or indicated a tricuspid aortic valve (GSE84274), as was also the case for healthy donors. In this context, the DMGs identified between the compared groups (TAA with BAV/IAA and TAA with BAV/ TAAD), even after adjustment for cell composition, may reflect DNA methylation features of aortic tissues determined by aortic valve morphology rather than by aortic dilatation per se. This is indirectly supported by the observation that the largest number of DMGs, obtained when comparing groups with a tricuspid valve (TAAD/IAA), does not reflect differences in DNA methylation patterns between diseased and intact aortic tissue with the same aortic valve morphology, since these differences disappear after adjustment for cell composition

DNA methylation analysis of aortic tissues conducted by other research groups has shown that, consistent with the present study, hypomethylated regions of the DIP2C, LMO4, NFIX, NKD1, and CYP2W1 genes are detected in patients with BAV, both in aneurysmal and in intact ascending aortic tissues (Björck et al., 2018). Moreover, significantly more hypomethylated regions are observed in ascending aortic tissues with bicuspid compared to tricuspid aortic valve, which is accompanied by a more active transcriptional profile in aortic tissues with BAV (Björck et al., 2018). At the same time, in atherosclerotic plaque of the aorta compared to intact aortic tissue from the same donors (where aortic valve morphology was not specified), hypermethylated CpG sites were identified in the DIP2C, NFIX, NKD1, and ARAP2 genes (Zaina et al., 2014).

Differences in aortic valve morphology also result in altered ascending aortic function, which are largely determined by the asymmetric distribution of blood flow velocity by BAV, contributing to a higher frequency of ascending aortic pathologies (Viscardi et al., 2010). Hemodynamic disturbances are the primary factor leading to alterations in the structural organization of the vascular tissue and in genomic functioning, resulting in the formation of distinct patterns of expressed genes, microRNAs, and proteins in patients with BAV and TAV, which however drive the development of the same phenotype (Folkersen et al., 2011; Kjellqvist et al., 2013; Gallo et al., 2018; Freiholtz et al., 2023). Differences in gene expression profiles between BAV and TAV groups in ascending aortic aneurysm have been identified both in ascending aortic tissues and in individual cell types, but the lists of differentially expressed genes do not include the DMGs identified in the present study (Lei et al., 2022; Doppler et al., 2023; Huang M. et al., 2023).

Genetic factors play an important role in the etiology of BAV, with heritability estimates ranging from 47 to 89 % (Gehlen et al., 2023). Association studies, GWAS, and analyses of rare clinically significant variants have led to the identification of more than forty genes associated with BAV development (Gago-Díaz et al., 2017; Chen S. et al., 2022; Gehlen et al., 2023; Carlisle et al., 2024; Sticchi et al., 2025). However, the genes identified in the present study are not among those associated with BAV development. Nevertheless, evidence suggests an association between the DMGs identified in the present study and cardiovascular diseases (CVD). For instance, the DIP2C gene is involved in transcription factor binding, and its deficiency leads to the development of congenital heart defects (Xia et al., 2025).

The NFIX and LMO4 genes encode transcription factors. Clinically significant variants of the NFIX gene are associated with thoracic aortic aneurysm and dissection in Malan syndrome (Oshima et al., 2017; Chevarin et al., 2020), whereas LMO4 is overexpressed in regions of turbulent blood flow, including the carotid arteries, the lesser curvature of the aortic arch, and the coronary arteries, suggesting a potential role for this gene in mechanosensitivity and pro-atherogenic response (Ni et al., 2010). The C10orf41 (ZNF503-AS2) gene encodes a long non-coding RNA (lncRNA). Increased methylation levels of the ZNF503-AS2 gene were detected in the plasma of patients with myocardial infarction following percutaneous coronary intervention compared to healthy donors (Ren et al., 2022).

PLEKHF1 is a lysosome-associated protein that induces apoptosis and autophagy. It is known that autophagy is closely associated with the onset and progression of atherosclerosis, myocardial ischemia/reperfusion, heart failure, and aortopathy in combination with BAV (Forte et al., 2019; Jiang B. et al., 2023). PLEKHF1 is one of eight genes associated with overall survival in dilated cardiomyopathy, and its expression is reduced in myocardial tissues of patients compared to the control group (Zhang et al., 2022). Overall, the PLEKHF1 gene is considered a potential biomarker for the development of CAD in patients with T2DM and cardiomyopathy (Huang Q. et al., 2020; Zhang et al., 2022).

The protein product of the NKD1 gene is an inhibitor of the Wnt signaling pathway, which is essential for the generation of cardiac progenitor cells, neural crest cell migration and differentiation, and the formation of the outflow tract and heart valves (Weerackoon et al., 2021). Dysregulation of the Wnt signaling pathway is one of the factors influencing the development of cardiovascular diseases, including structu- ral abnormalities of the aorta, which is directly associated with a predisposition to aneurysms (Pozo-Vilumbrales et al., 2025).

Variants of the transcription factor gene TBX5, which is essential for the formation of the atrial and ventricular septa and the cardiac conduction system, are associated with the development of congenital heart defects, Holt–Oram and Brugada syndromes, atrial fibrillation, acute myocardial infarction, and BAV (Steimle, Moskowitz, 2017; Wang F. et al., 2017; Wang S. et al., 2019; Jiang W.F. et al., 2020). TBX3 is also a transcription factor important for the differentiation of the sinoatrial and atrioventricular nodes. Variants of the TBX3 gene are associated with cardiac conduction abnormalities, the development of atrioventricular block, hypertrophic cardiomyopathy, atrial fibrillation, and heart failure (Lakda et al., 2025).

ARAP2 is a protein that activates the Arf6 GTPase and is involved in the regulation of focal adhesion, remodeling of actin fibers, and integrin recycling in vascular cells (Vitali et al., 2019). Altered ARAP2 gene activity in the aorta in mouse models (Eln−/− line) reflects modulation of the cellular cytoskeleton and intercellular interactions in the aortic vascular wall under conditions of severe elastic fiber defects (Staiculescu et al., 2018). In humans, the single nucleotide variant rs116215256 of the ARAP2 gene is associated with cardiac troponin I levels, which is known to be a regulator of myocardial contraction (Yang et al., 2021).

The PCDH7 gene encodes protocadherin 7, a membrane protein involved in cell recognition and cell–cell adhesion. The cadherin family plays a key role in morphogenesis as well as in the development of various pathological conditions, including cardiovascular diseases such as dilated cardiomyopathy, atherosclerosis, and restenosis, which involve significant remodeling of tissue architecture leading to clinical manifestations of disease (Joshi et al., 2024).

The MED13L gene encodes one of the subunits of the large mediator complex, which is involved in regulating the transcriptional activity of genes in various signaling pathways during early development, including heart development (Henry et al., 2025). Pathogenic variants in the MED13L gene have been associated with transposition of the great arteries, coarctation of the aorta, and other cardiovascular diseases (Schiano, Napoli, 2025).

Thus, the genes identified in the present study play important roles in the development of the cardiovascular system, and disruption of their function may lead to the development of cardiovascular diseases. The association of these genes with aortic dilatation/aneurysm in patients with bicuspid aortic valve through alterations in their epigenetic regulation in diseased aortic tissues is reported for the first time in the present study.

## Conclusion

Assessment of cellular composition based on DNA methylation profiles in aortic tissue samples allows us to evaluate the presence and extent of inflammation, as determined by the degree of immune cell infiltration into the arterial wall. This approach also characterizes the role of the inflammatory infiltrate in disrupting aortic wall integrity, as well as delineates genes that reflect differences associated with the studied phenotypes rather than sample cellular heterogeneity.

The genes NFIX, PCDH7, MED13L, LMO4, DIP2C, PLEKHF1, NKD1, C10orf41 (ZNF503-AS2), ARAP2, TBX3, and TBX5 may be considered novel candidate genes for the development of aortic dilatation/aneurysm in patients with bicuspid aortic valve. Particular attention should be paid to the genes NFIX, LMO4, DIP2C, C10orf41 (ZNF503-AS2), MED13L, TBX3, and TBX5, which encode transcription factors or regulators of DNA transcriptional activity, as well as the gene NKD1, an inhibitor of the Wnt signaling pathway.

Furthermore, the obtained data once again underscore the need for a rigorous approach to forming comparison groups with a clear and complete description of the studied phenotype, as well as for conducting studies at the single-cell level.

## Conflict of interest

The authors declare no conflict of interest.
